# Towards Explainable Artificial Intelligence for GNSS Multipath LSTM Training Models

**DOI:** 10.3390/s25030978

**Published:** 2025-02-06

**Authors:** He-Sheng Wang, Dah-Jing Jwo, Zhi-Hang Gao

**Affiliations:** Department of Communications, Navigation, and Control Engineering, National Taiwan Ocean University, 2 Peining Road, Keelung 202301, Taiwan; hswang@email.ntou.edu.tw (H.-S.W.); 0076c016@mail.ntou.edu.tw (Z.-H.G.)

**Keywords:** GNSS, multipath, explainability, layer-wise relevance propagation, long short-term memory

## Abstract

This paper addresses the critical challenge of understanding and interpreting deep learning models in Global Navigation Satellite System (GNSS) applications, specifically focusing on multipath effect detection and analysis. As GNSS systems become increasingly reliant on deep learning for signal processing, the lack of model interpretability poses significant risks for safety-critical applications. We propose a novel approach combining Recurrent Neural Networks (RNNs) with Long Short-Term Memory (LSTM) cells with Layer-wise Relevance Propagation (LRP) to create an explainable framework for multipath detection. Our key contributions include: (1) the development of an interpretable LSTM architecture for processing GNSS observables, including multipath variables, carrier-to-noise ratios, and satellite elevation angles; (2) the adaptation of the LRP technique for GNSS signal analysis, enabling attribution of model decisions to specific input features; and (3) the discovery of a correlation between LRP relevance scores and signal anomalies, leading to a new method for anomaly detection. Through systematic experimental validation, we demonstrate that our LSTM model achieves high prediction accuracy across all GNSS parameters while maintaining interpretability. A significant finding emerges from our controlled experiments: LRP relevance scores consistently increase during anomalous signal conditions, with growth rates varying from 7.34% to 32.48% depending on the feature type. In our validation experiments, we systematically introduced signal anomalies in specific time segments of the data sequence and observed corresponding increases in LRP scores: multipath parameters showed increases of 7.34–8.81%, carrier-to-noise ratios exhibited changes of 12.50–32.48%, and elevation angle parameters increased by 16.10%. These results demonstrate the potential of LRP-based analysis for enhancing GNSS signal quality monitoring and integrity assessment. Our approach not only improves the interpretability of deep learning models in GNSS applications but also provides a practical framework for detecting and analyzing signal anomalies, contributing to the development of more reliable and trustworthy navigation systems.

## 1. Introduction

GNSS systems serve as a critical infrastructure for modern positioning, navigation, and timing services. As these systems become increasingly integrated into safety-critical applications such as autonomous vehicles, precision agriculture, and aviation, ensuring their reliability and accuracy becomes paramount. One of the most significant challenges in GNSS operation is multipath interference, where signals reach receivers through multiple paths due to reflections from surrounding structures. This phenomenon can significantly degrade positioning accuracy and reliability, particularly in urban environments.

Recent advances in artificial intelligence, particularly deep learning, have shown promising results in addressing GNSS multipath challenges. However, the adoption of deep learning models in safety-critical GNSS applications faces a crucial obstacle: the lack of interpretability. While these models can achieve high accuracy in multipath detection and mitigation, their black-box nature makes it difficult to understand and validate their decision-making processes. This limitation poses significant risks in applications where system failures could have severe consequences.

The present study addresses three fundamental challenges in the application of deep learning to GNSS multipath detection:

- How can we develop deep learning models that not only detect multipath effects accurately but also provide interpretable results?
- How can we validate the decision-making process of these models in a way that builds trust in their predictions?
- How can we leverage model interpretability to enhance anomaly detection in GNSS signals?

To address these challenges, we propose a novel framework that combines RNNs using LSTM cells with LRP. Our approach not only achieves high accuracy in multipath detection but also provides transparent insights into the model’s decision-making process. We demonstrate this framework using real-world GNSS data collected from a Trimble BD970 dual-frequency receiver, focusing on scenarios where multipath effects are particularly challenging to detect and interpret.

Our key contributions include:

- The development of an interpretable deep learning architecture specifically designed for GNSS multipath detection.
- The adaptation and implementation of LRP techniques for analyzing temporal dependencies in GNSS signals.
- The discovery of a correlation between LRP relevance scores and signal anomalies, leading to a new method for anomaly detection.
- Comprehensive evaluation using real-world GNSS data, demonstrating both the accuracy and interpretability of our approach.

The development of GNSS multipath detection and mitigation techniques has evolved significantly over the past decades. Early research focused primarily on traditional signal processing approaches. In 2013, Phan et al. [1] proposed a nonlinear regression approach using Support Vector Machine (SVM) to estimate multipath effects based on satellite elevation and azimuth angles. Their work demonstrated the potential of machine learning in GNSS applications, achieving improved accuracy compared to conventional methods.

A significant advancement came in 2018 when Quan et al. [2] introduced a Convolutional Neural Network (CNN)-based method for multipath detection. Their approach could detect approximately 80% of multipath-affected data, marking a substantial improvement in detection capabilities. Building upon this progress, in 2020, Lin [3] proposed an LSTM-based multipath effect model that achieved remarkable accuracy rates of around 97% in prediction tasks. Most recently, Siemuri et al. [4] conducted a comprehensive review of the application of machine learning in the GNSS use case. Iqbal et al. [5] investigated deep learning for GNSS spoofing detection. An attention-based method for the GNSS jamming detection was introduced in [6].

In parallel with developments in multipath detection, significant advances have been made in GNSS interference monitoring and classification. Guo et al. [7] demonstrated the effectiveness of deep learning-based approaches for interference detection using time-frequency analysis. Recent work by Silva Lorraine et al. [8] has established comprehensive frameworks for network-based GNSS interference monitoring, while Jiang et al. [9] have shown how transfer learning can enable real-time interference classification. In [10], Mehr and Dovis proposed a CNN-based method for GNSS jamming detection and classification. These developments in interference detection share common challenges with multipath mitigation, particularly in terms of real-time processing requirements and the need for reliable detection methods. Furthermore, Zuo et al. [11] highlighted the importance of feature extraction and interpretation in interference detection systems, emphasizing the growing need for explainable AI approaches in GNSS signal quality monitoring.

Parallel to these developments in GNSS applications, the field of explainable artificial intelligence has gained increasing attention. While much of the early work in explainable AI focused on classification tasks, a distinct research field has emerged addressing the unique challenges of explaining regression models. In 2022, Letzgus et al. [12] published a groundbreaking paper in the IEEE Signal Processing Journal, proposing methods to apply XAI principles to regression models, opening new possibilities for GNSS applications. In [13], the authors also provided a comprehensive overview of the XAI technique. Unlike classification problems where feature importance can be directly linked to discrete class decisions, regression problems require different approaches to interpret continuous output predictions. This distinction is particularly relevant for GNSS applications, where the primary task involves predicting continuous values for signal parameters and error estimates.

The evolution of deep learning interpretability has seen various approaches. Layer-wise Relevance Propagation (LRP), introduced by Bach et al. [14] in 2015, has emerged as a powerful technique for explaining neural network decisions. Recent work by Arras et al. [15] has extended LRP to recurrent neural networks, including LSTM architectures, making it particularly relevant for time-series analysis in GNSS applications. In 2019, Montavon et al. [16] provided a detailed review of the LRP method.

As deep learning and artificial intelligence systems become increasingly prevalent, concerns about their transparency and interpretability have grown significantly. These concerns include potential threats like negative side effects, reward hacking, scaling oversight, safe exploration, and robustness to distribution shifts [17].

The field of XAI for regression has developed several specialized approaches. Local Interpretable Model-agnostic Explanations (LIME) for regression, introduced by Ribeiro et al. [18], provides local approximations of complex models using interpretable linear models. However, these approximations often struggle with temporal dependencies, making them less suitable for time-series applications like GNSS signal processing.

The second approach focuses on local perturbation analysis. This category includes gradient-based methods [19], which have a long history in deep learning interpretation. Sensitivity Analysis (SA) represents a prominent example of this approach [20,21,22], though it faces challenges such as gradient shattering and explanation discontinuities. Recent advancements by Montavon et al. [23] and Smilkov et al. [24] have addressed these limitations through techniques like regional average gradients and specific path integration methods.

Perturbation-based and optimization-based methods form another subset of this approach. Zeiler and Fergus [25] demonstrated the effectiveness of masking input regions to test feature importance. Prediction Difference Analysis (PDA) [26] advanced this concept by employing conditional sampling within feature regions. Meaningful Perturbation [27,28] and Activation Maximum [29] represent further developments in this direction, though their optimization requirements increase computational complexity.

The third major approach involves a propagation-based explanation, with LRP emerging as a leading technique. Bach et al. [14] and Montavon et al. [16] introduced LRP, which integrates explanation processes with model structure and has demonstrated effectiveness across various architectures, including deep neural networks, LSTMs [30], and Fisher vector classifiers [31]. Recent work has extended LRP’s application to anomaly detection through neuralization [32,33,34].

LRP has evolved to include several variants. The Basic Rule (LRP-0) provides proportional relevance distribution based on neuron weights. The Epsilon Rule (LRP-ε) introduces numerical stability for handling small or contradictory contributions. The Gamma Rule (LRP-γ) offers enhanced control over positive and negative contributions, with parallels to other techniques like DeepLIFT [35] and Top-down neural attention [36].

Recent research has particularly focused on applying these explainability techniques to regression problems, an area that has received less attention compared to classification tasks [12]. This evolution in explainable AI research reflects a growing recognition of the importance of understanding AI decision-making processes, particularly in critical applications where transparency and accountability are essential.

The literature reveals a clear trend toward integrating explainability directly into model architectures rather than treating it as a post-hoc analysis. This integration allows for more robust and reliable interpretations while potentially improving model performance through better understanding of internal decision mechanisms.

Recent advances have specifically addressed time-series regression problems. Wu et al. [37] demonstrated the application of Layer-wise Relevance Propagation to LSTM-based regression models in predictive maintenance, showing how relevance scores can be meaningfully interpreted for continuous predictions. Building on this work, Kauffmann et al. [32] developed techniques for explaining anomalies in regression outputs, particularly relevant for our investigation of GNSS signal anomalies. In the context of GNSS security and reliability, Rathod et al. [38] in the study of pattern detection, demonstrate how explainable AI can enhance the robustness of deep learning models in critical applications.

As the reliance on AI-driven systems increases in critical applications such as autonomous navigation and precision timing, there is a growing need for explainable artificial intelligence (XAI) in the field of GNSS. XAI aims to make the decisions and predictions of AI models more transparent and interpretable to humans, which is crucial for ensuring the reliability, safety, and trustworthiness of these systems.

This study focuses on developing explainable AI techniques for LSTM-based GNSS multipath models. By incorporating LRP and other interpretability methods, we aim to provide insights into how these models process GNSS data and make predictions about multipath effects. This research not only contributes to the advancement of GNSS technology but also addresses the broader challenge of making complex AI systems more understandable and accountable.

The objectives of this study are to:

1. Implement an LSTM-based model for GNSS multipath prediction.
2. Apply LRP and other XAI techniques to interpret the model’s decision-making process.
3. Analyze the relevance of different input features in multipath prediction.
4. Evaluate the model’s behavior under normal and anomalous conditions.
5. Discuss the implications of explainable AI for improving GNSS reliability and integrity.

By achieving these objectives, this research aims to bridge the gap between the high performance of deep learning models and the need for transparency in critical GNSS applications. The findings of this study have the potential to enhance the robustness of GNSS systems against multipath effects and pave the way for more trustworthy AI-driven navigation solutions.

The remainder of this paper is organized as follows: Section 2 presents a brief theoretical background of multipath estimation in GNSS and describes our RNN architecture using LSTM cells and its implementation. Section 3 details the adaptation of LRP for GNSS applications. Section 4 presents our experimental results, followed by discussion and conclusions in Section 5 and Section 6, respectively.

## 2. Background

### 2.1. GNSS Multipath Effect

Multipath effects occur when GNSS signals reach a receiver through multiple paths due to reflections from surrounding objects. This phenomenon is particularly pronounced in urban environments where buildings and other structures create complex reflection patterns. The interaction between direct and reflected signals can significantly degrade positioning accuracy, making multipath detection and mitigation crucial for reliable GNSS applications.

The impact of multipath can be quantified through two fundamental GNSS measurements: pseudorange (code phase) and carrier phase observations. The pseudorange measurement *P_i_* for carrier frequency *f_Li_* (*i* = 1, 2) includes various error components:

$$\begin{array}{l} P_{1}=r+I_{1}+T+M_{1}+c(dt_{r}-dt^{s})\\ P_{2}=r+I_{2}+T+M_{2}+c(dt_{r}-dt^{s}) \end{array}$$

where

- *P_i_* is code-phase observable for carrier frequency *f_Li_* (*i* = 1, 2);
- *r* is the geometric distance;
- *I*_1_, *I*_2_ are ionospheric delay errors;
- *T* is the tropospheric delay error;
- *M*_1_, *M*_2_ are pseudorange multipath errors;
- *c* is the speed of light;
- *dt_r_* is the receiver clock error;
- *dt^s^* is the satellite clock error.

Carrier phase measurements provide more precise observations but include an unknown integer ambiguity term:

$$\begin{array}{l} L_{1}=r-I_{1}+T+m_{1}+n_{1}\lambda_{1}+c(dt_{r}-dt^{s})\\ L_{2}=r-I_{2}+T+m_{2}+n_{2}\lambda_{2}+c(dt_{r}-dt^{s}) \end{array}$$

where:

- *m*_1_, *m*_2_ are carrier phase multipath errors;
- *n*_1_, *n*_2_ are the integer ambiguity terms;
- λ_1_, λ_2_ are the carrier wavelengths.

The multipath effect in GNSS occurs when satellite signals reach the receiver through multiple paths due to reflection and diffraction from surrounding objects. To estimate multipath errors, we utilize both pseudorange and carrier phase measurements from dual-frequency observations. Following the methodology proposed by Estey and Meertens [39], the multipath equations can be derived through linear combinations of these measurements. For dual-frequency observations, assuming the atmospheric path delay is almost identical for both frequencies, the carrier phase linear combinations can be expressed as:

$$I_{1}+\frac{1}{\alpha -1}(n_{1}\lambda_{1}-n_{2}\lambda_{2}+m_{1}-m_{2})=\frac{1}{\alpha -1}(L_{1}-L_{2})$$

or

$$I_{2}+\frac{\alpha }{\alpha -1}(n_{1}\lambda_{1}-n_{2}\lambda_{2}+m_{1}-m_{2})=\frac{\alpha }{\alpha -1}(L_{1}-L_{2})$$

where:

- *L*_1_, *L*_2_ are the carrier phase measurements;
- *I*_1_, *I*_2_ represents the ionospheric delay errors;
- *n*_1_, *n*_2_ are the integer ambiguities;
- *m*_1_, *m*_2_ are the phase multipath errors;
- α is the ratio related to dual frequencies.

The ionospheric delay error ratio α between dual frequencies can be expressed as:

$$\alpha =\frac{f_{L1}^{2}}{f_{L2}^{2}}$$

To isolate multipath effects, we can form linear combinations of these measurements. The resulting multipath combination *mp_i_* can be expressed as:

$$\begin{array}{l} mp_{1}=P_{1}-\left(1+\frac{2}{\alpha -1}\right)L_{1}+\left(\frac{2}{\alpha -1}\right)L_{2}=M_{1}+B_{1}-\left(1+\frac{2}{\alpha -1}\right)m_{1}+\left(\frac{2}{\alpha -1}\right)m_{2}\\ mp_{2}=P_{2}-\frac{2\alpha }{\alpha -1}L_{1}+\left(\frac{2\alpha }{\alpha -1}-1\right)L_{2}=M_{2}+B_{2}-\left(\frac{2\alpha }{\alpha -1}\right)m_{1}+\left(\frac{2\alpha }{\alpha -1}-1\right)m_{2} \end{array}$$

where:

- *mp*_1_, *mp*_2_ are the multipath combinations;
- *P*_1_, *P*_2_ are the pseudorange measurements;
- *M*_1_, *M*_2_ represent the multipath errors;
- *B*_1_, *B*_2_ are the bias terms related to integer ambiguities.

The bias terms can be expressed as:

$$\begin{array}{l} B_{1}=-\left(1+\frac{2}{\alpha -1}\right)n_{1}\lambda_{1}+\left(\frac{2}{\alpha -1}\right)n_{2}\lambda_{2}\\ B_{2}=-\left(\frac{2\alpha }{\alpha -1}\right)n_{1}\lambda_{1}+\left(\frac{2\alpha }{\alpha -1}-1\right)n_{2}\lambda_{2} \end{array}$$

To obtain the multipath error variations, we subtract the mean multipath value over time from the multipath combinations:

$$MP_{1}=mp_{1}-mp_{1t}\;\mathrm{and}\;MP_{2}=mp_{2}-mp_{2t},$$

where *mp*_1*t*_ and *mp*_2*t*_ are the time-averaged multipath values:

$$mp_{1t}=\frac{1}{t}\sum_{t}mp_{1}\mathrm{and}\;mp_{2t}=\frac{1}{t}\sum_{t}mp_{2}.$$

While these fundamental principles describe the multipath phenomenon, effectively detecting and analyzing these effects in real-time requires advanced computational approaches. Deep learning, particularly recurrent neural networks with LSTM cells, offers promising solutions for processing such temporal data.

### 2.2. Deep Learning for Time Series Analysis

Our choice of RNNs with LSTM cells for this study is driven by several key considerations, particularly the balance between model capability and explainability. While more recent architectures such as Gated Recurrent Units (GRU), Minimal Gated Units (MGU), and Transformers have demonstrated superior performance in various sequence modeling tasks [6], LSTM cells offer distinct advantages for our specific investigation into explainable AI for GNSS applications.

First, LSTM cells are particularly well suited for capturing the temporal dependencies inherent in GNSS multipath effects. The cell’s memory mechanism can maintain relevant information over extended sequences, which is crucial for tracking gradual changes in multipath patterns. While Transformer architectures excel at modeling long-range dependencies through self-attention mechanisms, their complexity poses additional challenges for interpretation and real-time implementation in GNSS systems.

Second, the established theoretical framework for Layer-wise Relevance Propagation (LRP) with LSTM cells provides a solid foundation for our explainability investigation. The well-understood gating mechanisms in LSTM cells allow for the clearer interpretation of how the model processes temporal information, making it easier to attribute relevance scores to specific features and time steps. More recent architectures, while potentially offering better performance, lack the same level of theoretical development in terms of explainability techniques.

Third, practical considerations for GNSS applications influenced our choice. LSTM cells offer a good balance between computational efficiency and model capability, making them suitable for potential real-time implementation in GNSS receivers. Additionally, their simpler architecture compared to Transformers facilitates easier deployment on embedded systems with limited computational resources.

Alternative architectures such as Convolutional Neural Networks (CNNs) were also considered, as they could potentially capture spatial patterns in GNSS signals. However, our focus on temporal dependencies and the sequential nature of multipath effects made RNNs with LSTM cells a more appropriate choice. Future work could explore hybrid architectures combining CNNs for spatial feature extraction with recurrent cells for temporal pattern recognition.

It is worth noting that while architectures like GRU might offer similar capabilities with fewer parameters, and Transformers might provide better performance on certain tasks, our primary goal was to investigate model explainability rather than maximizing prediction accuracy. The LSTM cell’s well-understood architecture and established explainability techniques made it an ideal candidate for developing and demonstrating interpretable AI methods for GNSS applications.

**Figure 1:**
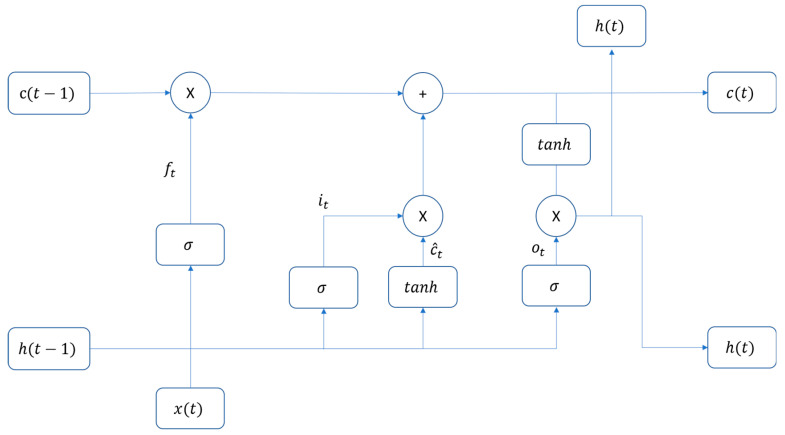
Standard architecture for an LSTM memory cell.

LSTM-based RNN, first introduced by Hochreiter and Schmidhuber [40] in 1997, represents a specialized form of recurrent neural networks designed to address the vanishing gradient problem in traditional RNNs. In 2024, Feng et al. [41] provided a state-of-the-art review of the LSTM and its future trend. The LSTM architecture incorporates sophisticated gating mechanisms that allow it to learn long-term dependencies in sequential data effectively. The standard architecture of an LSTM memory cell is illustrated in Figure 1, showing the key components including the memory cell state, forget gate, input gate, and output gate. This architecture enables the network to effectively learn and maintain relevant information over time. Each gate serves a specific purpose in controlling information flow through the network. The key equations governing LSTM cell operation are:

$$f_{t}=\sigma (W_{f}x_{t}+U_{f}h_{t-1}+b_{f})\;\text{//Forget gate}\;i_{t}=\sigma (W_{i}x_{t}+U_{i}h_{t-1}+b_{i})\;\text{//Input gate}\;{\hat{c}}_{t}=\tanh(W_{c}x_{t}+U_{c}h_{t-1}+b_{c})\;\text{//Candidate values}\;c_{t}=f_{t}⊙c_{t-1}+i_{i}⊙{\hat{c}}_{t}\;\text{//Cell state update}\;o_{t}=\sigma (W_{o}x_{t}+U_{o}h_{t-1}+b_{o})\;\text{//Output gate}\;h_{t}=o_{t}⊙\tanh(c_{t})\;\text{//Hidden state}$$

where:

- σ is the sigmoid activation function;
- *W*-terms are weight matrices;
- *U*-terms are the weights of the recurrent connections between gates and the previous hidden state;
- *x*-terms are current inputs;
- *b*-terms are biases;
- ⊙ denotes element-wise multiplication (Hadamard product).

This architecture is particularly suitable for GNSS multipath detection because:

- It can capture long-term dependencies in signal patterns;
- The gating mechanism helps identify relevant temporal features;
- It can process variable-length sequences of multipath measurements;
- The cell state provides a memory mechanism for tracking signal evolution.

Understanding these fundamentals of multipath effects and LSTM cells is essential for appreciating how Layer-wise Relevance Propagation can be adapted to interpret deep learning models in GNSS applications, which we discuss in detail in Section 3.

Given these capabilities of deep learning for time series analysis, we next describe how we adapted and optimized the network architecture specifically for GNSS multipath detection.

### 2.3. Architecture Selection and Implementation

The LSTM architecture used in this study was designed to balance model complexity with computational efficiency while maintaining robust performance for GNSS multipath detection. The network configuration was determined through a systematic evaluation process, considering both empirical testing and insights from the related literature in GNSS applications.

The primary architecture consists of a single LSTM layer with 256 hidden units, followed by a dense output layer. This configuration was chosen after testing various architectures with hidden units ranging from 64 to 512. While deeper architectures with multiple LSTM layers were evaluated, the single-layer configuration with 256 units provided optimal performance without excessive computational overhead. The number of hidden units (256) proved sufficient to capture the temporal dependencies in GNSS signals while avoiding overfitting, as validated through our cross-validation experiments.

The model processes sequences of length 60 (window size), chosen based on the temporal correlation characteristics of GNSS multipath effects. This window size represents approximately one minute of data at our sampling rate, providing sufficient context for the model to learn relevant patterns while maintaining computational efficiency. The batch size of 32 was selected as a compromise between training stability and memory usage, following common practice in deep learning applications.

The learning rate of 0.000025 was determined through a grid search over the range [1 × 10^−6^, 1 × 10^−2^]. This relatively small learning rate ensures stable convergence given the sensitivity of GNSS measurements. We employed the Adam optimizer with default beta parameters (β_1_ = 0.9, β_2_ = 0.999), as it demonstrated superior convergence characteristics compared to standard SGD in our preliminary experiments.

**Figure 2:**
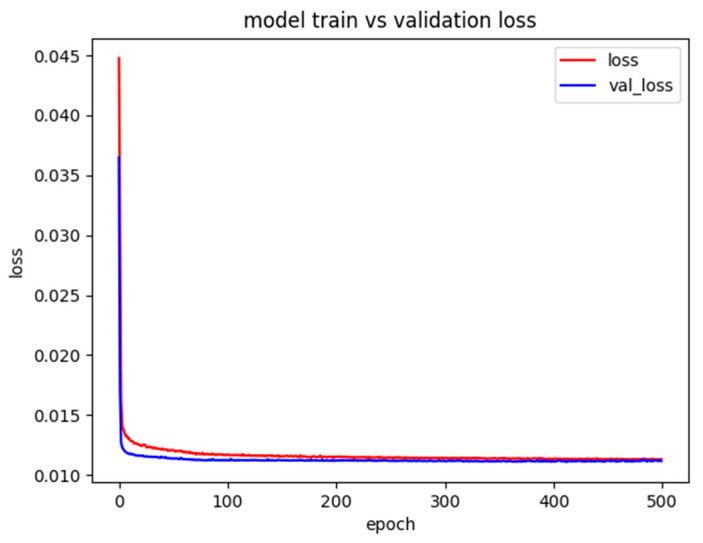
Training and validation loss curves over 500 epochs for the LSTM model. The convergence behavior shows that training stabilizes around 400 epochs, with both losses decreasing steadily and minimal gap between training and validation curves, indicating good generalization without overfitting. The additional 100 epochs ensure stable optimization and model robustness.

The number of training epochs (500) was established through convergence analysis. Figure 2 shows the learning curves for both training and validation loss, indicating that the model typically converges around 400 epochs, with the additional 100 epochs ensuring stable optimization.

The selected architecture was validated against several alternatives (Table 1). While larger models (512 units) showed marginally better validation MSE ($2.83\times 10^{-3}$ vs. $2.87\times 10^{-3}$), they required significantly more computational resources and training time. The dual-layer configuration (256 units per layer) performed similarly to our chosen architecture but with increased complexity and training time.

A dropout rate of 0.2 was implemented based on validation performance, providing regularization without significantly impacting model capacity. This value was selected after testing rates between 0.1 and 0.5, with 0.2 offering the best trade-off between regularization and model performance.

The selected architecture requires approximately 312 MB of GPU memory during training, making it feasible to deploy on most modern hardware. The model contains 329,728 trainable parameters, which is relatively modest for deep learning applications while sufficient for our task. During inference, the model processes a single time step in approximately 2.3 ms on standard GPU hardware, making it suitable for real-time applications.

This architecture represents a practical compromise between model complexity and performance, prioritizing robust performance and interpretability over marginal improvements in accuracy that might come from more complex configurations. The relatively simple architecture also facilitates the application of Layer-wise Relevance Propagation, as fewer layers reduce the complexity of relevance propagation paths.

With this understanding of GNSS multipath effects and our chosen deep learning approach, we now present our core methodological contribution: the adaptation and implementation of LRP for interpreting LSTM-based GNSS multipath detection.

## 3. Methodology: LRP for LSTM-Based GNSS Multipath Detection

### 3.1. Brief Introduction to LRP

LRP represents a significant advancement in making deep neural networks more interpretable and transparent. Originally proposed by Bach et al. [14] in 2015, LRP is a technique designed to decompose the predictions of deep neural networks by calculating the contributions of individual input features to the final output, effectively tracing back the network’s decision-making process layer by layer.

The fundamental principle of LRP is based on a conservation law where the total relevance score distributed to one layer must equal the total relevance distributed in the previous layer. Mathematically, this can be expressed as:

$$\sum_{i}R_{i}(m)=\sum_{j}R_{j}(n)=f(x)$$

where *R_i_*(*m*) and *R_j_*(*n*) are the relevance scores of individual neurons *i*, *j* in layers *m* and *n*, respectively. The form of *f*(*x*) will be defined later on.

LRP implements several rules to govern how relevance scores propagate between layers in order to fit the characteristics of different neural networks architecture:

1. Basic Rule (LRP-0):

$$R_{i}(m)=\sum_{j}\left(\frac{z_{ij}}{\sum_{k}z_{kj}}\right)R_{j}(n)$$

where *z_ij_* is the amount of input contributed by activated neuron *i* in layer *m* to neuron *j* in layer *n*; $\sum_{k}z_{kj}$ is the total amount of contribution sent to neuron *j* from all connected neurons in layer *m* before a non-linear activation function is applied. This rule redistributes relevance based on the proportional contributions of each neuron.

2. Epsilon Rule (LRP-ε):

$$R_{i}(m)=\sum_{j}\left(\frac{z_{ij}}{\varepsilon +\sum_{k}z_{kj}}\right)R_{j}(n)$$

This variant introduces a small stabilizing term ε to handle numerical instabilities and small contributions.

3. Gamma Rule (LRP-γ):

LRP-γ is a variant of the Layer-wise Relevance Propagation algorithm specifically designed to handle neural networks with strong nonlinearities. In the gamma rule, relevance distribution is modulated by a parameter γ that controls the emphasis placed on positive contributions in the network. The rule is formulated as:

$$R_{i}(m)=\sum_{j}\left(\frac{z_{ij}+\gamma z_{ij}^{+}}{\sum_{k}\left(z_{kj}+\gamma z_{kj}^{+}\right)}\right)R_{j}(n)$$

where $z_{ij}^{+}$ refer to the amount of positive inputs that neuron *j* contributed from the higher layer *m*, and γ is a parameter to control the weights of positive contribution. This approach helps stabilize the relevance propagation by emphasizing positive contributions while suppressing negative ones, thereby producing more interpretable results. The gamma rule is particularly useful in deep architectures like LSTM-based RNNs where the traditional LRP rules might struggle with vanishing or exploding relevance scores.

When applied to complex architectures like LSTM-based RNNs, LRP requires special handling for multiplicative interactions and gated structures. For such cases, the “signal-take-all” strategy is often employed, where relevance flows through the signal path while gates control but do not carry relevance themselves. The significance of LRP lies in its ability to:

- Provide detailed insights into neural network decision-making;
- Identify key features contributing to specific predictions;
- Enable validation of model behavior;
- Support model improvement and optimization;
- Enhance trust in AI systems through transparency.

LRP has been successfully applied across various domains, including image classification, text analysis, and time-series prediction, demonstrating its versatility and effectiveness in making deep learning models more interpretable and trustworthy. Through the careful propagation of relevance scores, LRP creates a comprehensive explanation framework that bridges the gap between the black-box nature of deep neural networks and the need for interpretable AI systems in critical applications.

### 3.2. Implementation of LRP for LSTM-Based RNNs

After understanding the theoretical framework of both LSTM-based RNNs and Layer-wise Relevance Propagation, we now present a systematic approach to implementing LRP specifically for LSTM architectures. The implementation must address the unique characteristics of LSTM-based RNNs while maintaining the core principles of relevance conservation. For this purpose, we first introduce some notation. Let *s*, *g* be the neurons representing the signal and the gate, let *p* be the neuron representing the product of these two quantities. Let *f* be the neuron corresponding to the forget gate. Let *k* be the neuron on which the signal is being accumulated. Let *k*−1, *p*−1, … be the same neurons at previous time steps. Let *a* be the output of the activation. LSTM forward pass can be decomposed into the following three elementary types of computation:

1. linear mappings $z_{s}=\sum_{j}a_{j}w_{js},{\;z}_{g}=\sum_{j}a_{j}w_{jg}$
2. gated interactions $a_{p}=\tanh(z_{s})\cdot \sigma (z_{g})$
3. accumulation $a_{k}=a_{f}\cdot a_{k-1}+a_{p}$

The implementation of LRP begins with recording all necessary activations during the forward pass. Algorithm 1 is the pseudocode for forward pass recording.
**Algorithm 1:** Forward Pass RecordingInput: LSTM model M, input sequence X  Output: Activation records A  Initialize activation storage A 
 For each time step *t*:  Record input gate activations: $i_{t}=\sigma (W_{i}x_{t}+U_{i}h_{t-1}+b_{i})$
 Record forget gate activations: $f_{t}=\sigma (W_{f}x_{t}+U_{f}h_{t-1}+b_{f})$
 Record cell state: $c_{t}=f_{t}⊙c_{t-1}+i_{t}⊙\tanh(W_{c}x_{t}+U_{c}h_{t-1}+b_{c})$
 Record output gate activations: $o_{t}=\sigma (W_{o}x_{t}+U_{o}h_{t-1}+b_{o})$
 Record hidden state: $h_{t}=o_{t}⊙\tanh(c_{t})$
 Store all activations in A  Return A

The relevance is initialized at the output layer and propagated backward, as illustrated in Algorithm 2:
**Algorithm 2:** Relevance InitializationInput: Final prediction *P*, target class *c*
 Output: Initial relevance *R*_0_

 If classification task:  Set *R*_0_[*c*] = 1 (for target class)  Set *R*_0_[*i*] = 0 (for all other classes)  Else if regression task:  Set *R*_0_ = *P*
 Return *R*_0_

Algorithm 3 is the core of the implementation which handles the propagation through LSTM cells. Unlike linear mappings with their straightforward summation structure, LSTM cells contain multiplicative interactions that make relevance propagation more challenging. To address this complexity, Arras et al. [15] introduced the signal-take-all redistribution rule,

(*R_g_*, *R_s_*) = (0, *R_p_*)

This kind of redistribution provides a systematic way to handle relevance attribution through these nonlinear gated interactions.

The “signal-take-all” approach addresses the key challenge of determining how relevance should flow through an LSTM cell’s gates, where the output is controlled by multiplicative interactions rather than simple weighted sums. This method acknowledges that while gates control the flow of information, the actual information content resides in the signal path.
**Algorithm 3:** LSTM Relevance PropagationInput: Recorded activations *A*, initial relevance *R*_0_
 Output: Input relevance *R*

 Initialize relevance storage *R*
 For each time step *t* in reverse order:  // Handle multiplicative interactions  For each gate *g* in (input, forget, output):  Apply signal-take-all strategy: 
*R_s_* = *R*_0_

*R_p_* = 0 
 // Handle cell state  Calculate state relevance:        $\begin{array}{l} a_{k}=a_{f}\cdot a_{k-1}+a_{p}\\ R_{k}=a_{p}\cdot c_{k} \end{array}$
    $R_{k-1}=a_{f}\cdot a_{k-1}\cdot c_{k}$
 For *τ* = 1 to *T*:    $\begin{array}{cc} R_{p-1} & =a_{f}\cdot a_{p-1}\cdot c_{k}\\ R_{p-2} & =(a_{f}\cdot a_{f-1})\cdot a_{p-2}\cdot c_{k}\\ & \vdots \\ R_{p-\tau } & =\left(\prod_{t=1}^{\tau }a_{f-t+1}\right)\cdot a_{p-\tau }\cdot c_{k} \end{array}$

 // Apply LRP rules for linear mappings  For each linear transformation:  Apply LRP-ε or LRP-αβ rule:     $R_{i}(m)=\sum_{j}\left(\frac{z_{ij}}{\varepsilon +\sum_{k}z_{kj}}\right)R_{j}(n)$
       $R_{i}(m)=\sum_{j}\left(\alpha \frac{z_{ij}^{+}}{\sum_{k}z_{kj}^{+}}-\beta \frac{z_{ij}^{-}}{\sum_{k}z_{kj}^{-}}\right)R_{j}(n)$

 Accumulate relevance scores  Return R

This implementation framework provides a structured approach to applying LRP to LSTM-based RNNs while maintaining computational efficiency and numerical stability. The modular design allows for easy adaptation to different LSTM architectures and application requirements.

Having established our methodological framework, we now evaluate its effectiveness through a series of experiments designed to test both prediction accuracy and interpretability.

## 4. Experimental Results

**Figure 3:**
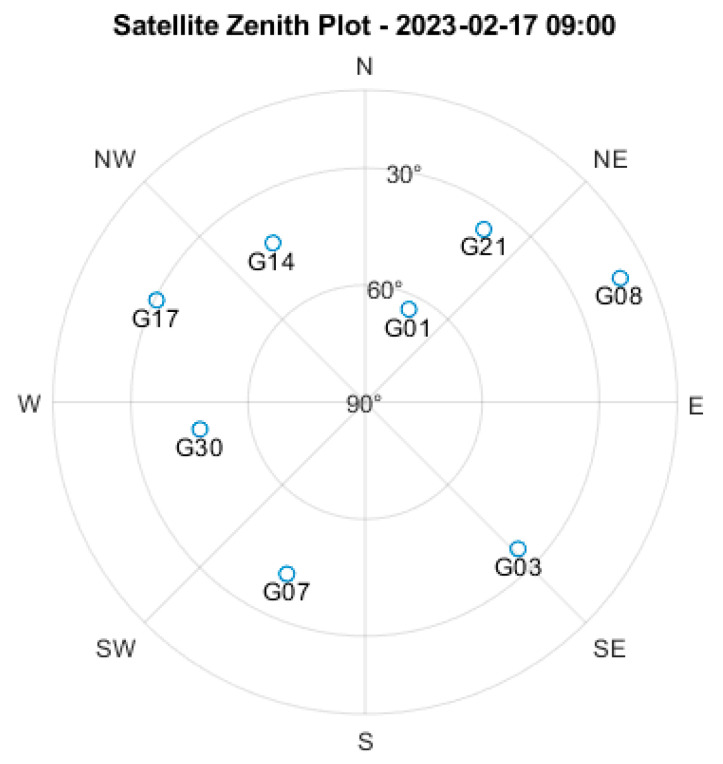
Skyplot showing satellite distribution at 9:00 a.m. on 17 February 2023. The G03 satellite (marked in the plot) was selected for analysis due to its low elevation angle, which makes it more susceptible to multipath effects in urban environments.

The experimental design and setup for analyzing GNSS multipath effects using LRP-interpreted LSTM-based RNNs was conducted using a Trimble BD970 dual-frequency GNSS receiver. The experimental setup focused on data collection from specific satellites, with particular attention to those at low elevation angles. Figure 3 shows the skyplot of satellite distribution at 9:00 a.m. on 17 February 2023, where the G03 satellite was selected for analysis due to its low elevation angle, making it more susceptible to multipath effects in urban environments. The receiver collected one second of vibration signal data every 10 min, with each snapshot containing 20,480 data points.

The LSTM model was designed to process five key features: L1 frequency multipath error variation (MP1), L2 frequency multipath error variation (MP2), L1 frequency carrier-to-noise ratio (CNR1), L2 frequency carrier-to-noise ratio (CNR2), and satellite elevation angle (EL). The network architecture was configured with 256 hidden units, using a learning rate of 0.000025 over 500 training epochs. The model processed data in batches of 32 samples, with a window size of 60 time steps. To prevent overfitting, a dropout rate of 0.2 was implemented, and 15% of the dataset was reserved for validation. The model was trained using Mean Square Error as the loss function. Configuration parameters of the LSTM are summarized in Table 2.

**Figure 4:**
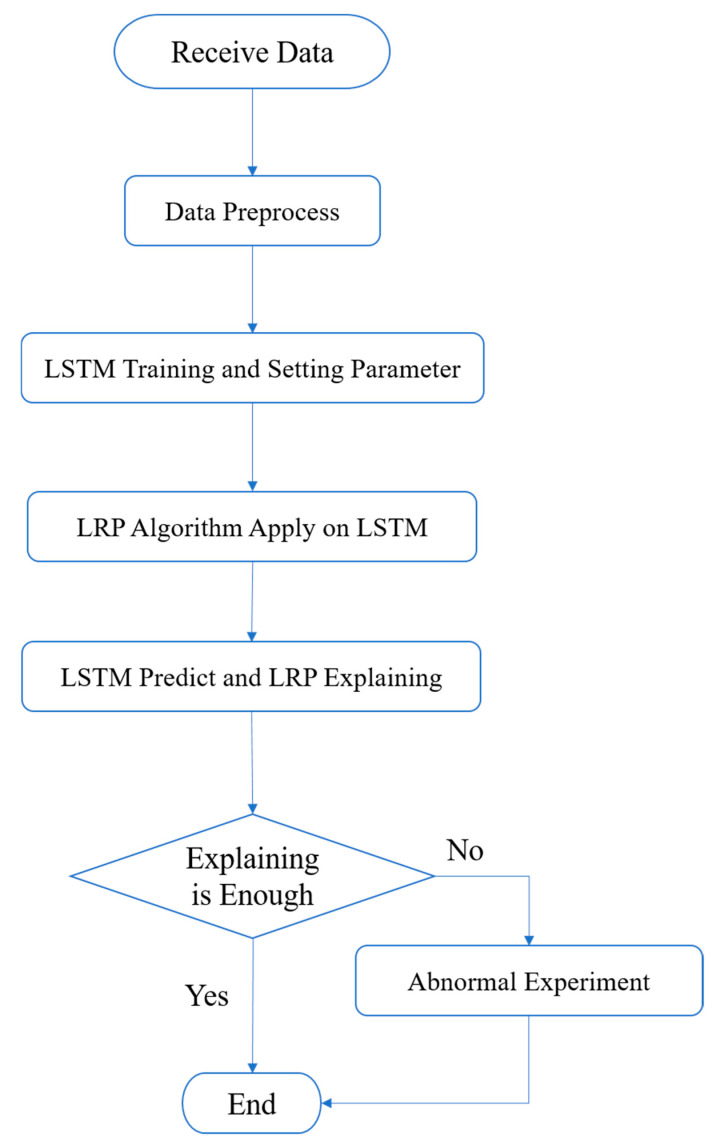
Experimental workflow diagram illustrating the sequence of data processing, model training, and analysis stages. The upper path shows the forward propagation process through the LSTM network layers, while the lower path demonstrates the backward propagation of relevance scores using LRP technique.

The flow chart of the experiment is shown in Figure 4. The experimental flow began with initial model training and validation using normal, non-anomalous data. After establishing baseline performance, the LRP algorithm was applied to analyze the trained model, generating relevance scores for each neuron corresponding to different features. Subsequently, three sets of experiments were conducted with varying periods of anomaly injection: from sample 1700 to 2000, 1400 to 2000, and 1100 to 2000. This progressive expansion of the anomaly period allowed for comprehensive analysis of the model’s response to different anomaly durations.

The evaluation process included both quantitative and qualitative analyses. Model performance was assessed through MSE calculations, while the effectiveness of LRP interpretation was evaluated through relevance score growth rates and distribution patterns. Visual analysis tools, including heat maps and distribution plots, were employed to examine the relationship between anomalies and relevance score changes. This comprehensive experimental approach enabled thorough evaluation of both the LSTM model’s predictive capabilities and the effectiveness of LRP in providing interpretable results for GNSS multipath detection.

**Figure 5:**
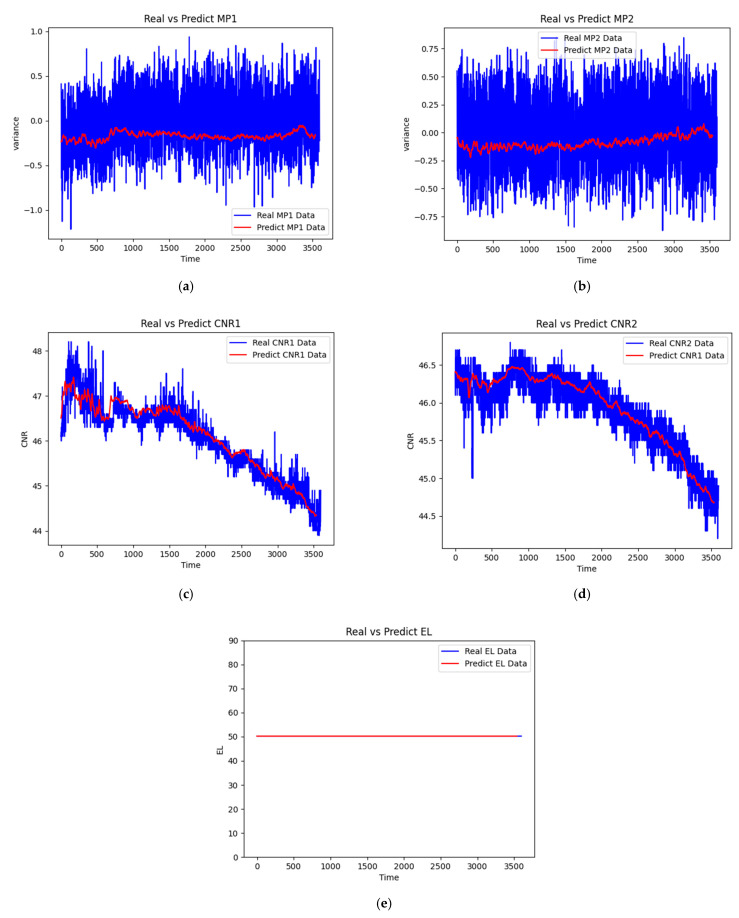
LSTM prediction results for GNSS observables. (**a**) First frequency multipath error variation (MP1), (**b**) second frequency multipath error variation (MP2), (**c**) first frequency carrier-to-noise ratio (CNR1), (**d**) second frequency carrier-to-noise ratio (CNR2), and (**e**) satellite elevation angle (EL). The blue lines represent actual measurements while red lines show the LSTM predictions. All parameters except EL demonstrate significant temporal variations, with the model achieving high prediction accuracy across different signal characteristics.

The prediction results from the LSTM model for different GNSS parameters are illustrated in Figure 5, demonstrating the model’s ability to predict both normal and anomalous conditions. The prediction results from the LSTM model for different GNSS parameters demonstrate good accuracy across all measured parameters. The model shows small prediction errors, with deviations between predicted and actual values being nearly indistinguishable in most cases. For multipath, the errors are usually well within 1 m, and, for CNR, the errors are with 1 dB/Hz.

In Figure 5a, the MP1 (first frequency multipath error variation) prediction results show strong agreement between the predicted and actual values. The model successfully captures both the general trend and local fluctuations in the multipath error, indicating its effectiveness in modeling first-frequency multipath patterns. Similarly, Figure 5b displays the MP2 (second frequency multipath error variation) predictions, which also demonstrate high accuracy in tracking the actual measurements, though with slightly different patterns from MP1 due to the frequency-dependent nature of multipath effects.

The carrier-to-noise ratio predictions shown in Figure 5c (CNR1) and Figure 5d exhibit particularly robust performance. The model accurately predicts the signal strength variations, which are crucial indicators of signal quality and potential multipath interference. The close alignment between predicted and actual values suggests that the LSTM-based RNN has effectively learned the underlying patterns in signal strength fluctuations.

While Figure 5e shows that the elevation angle (EL) remains relatively stable during the experiment period, its inclusion in the model proves to be valuable for a different reason. Although EL does not show significant temporal variation, subsequent analysis reveals its importance in explaining abnormal signal behaviors. The elevation angle serves as a crucial reference parameter that helps interpret multipath effects and signal anomalies, as demonstrated in the following sections of our analysis. This highlights that the significance of a parameter cannot be judged solely by its temporal variability but must also consider its role in the overall signal interpretation framework.

The minimal prediction errors across all parameters validate the effectiveness of our LSTM architecture and training approach, while also providing a reliable foundation for the subsequent LRP analysis.

**Figure 6:**
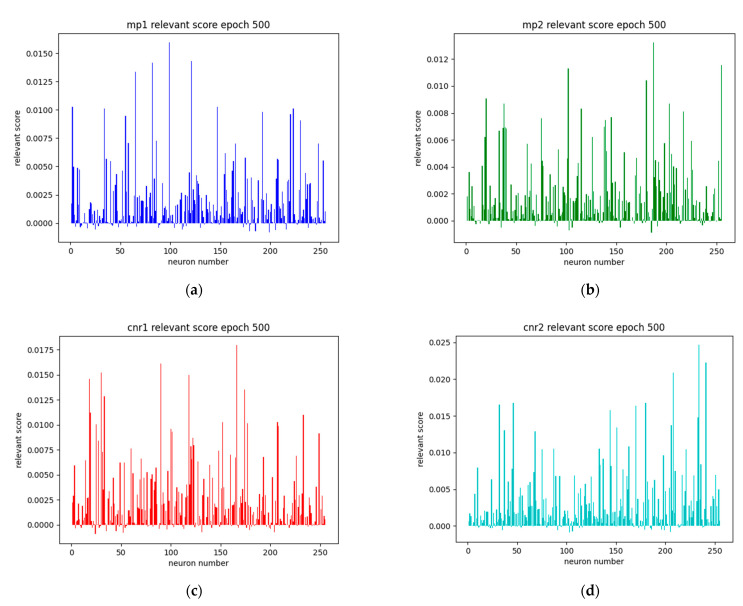

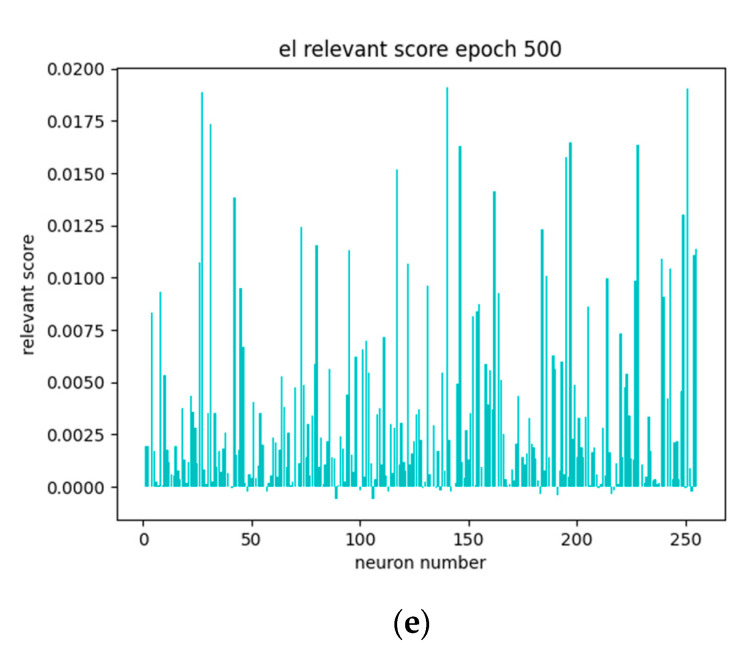
Distribution of LRP relevance scores across 256 neurons for different GNSS features under normal conditions: (**a**) first frequency multipath error variation (MP1), (**b**) second frequency multipath error variation (MP2), (**c**) first frequency carrier-to-noise ratio (CNR1), (**d**) second frequency carrier-to-noise ratio (CNR2), and (**e**) satellite elevation angle (EL). The relevance scores represent each neuron’s contribution to the model’s prediction, calculated using the LRP-gamma algorithm with γ = 2.5.

To analyze the model’s decision-making process, we examined the distribution of LRP relevance scores across the network’s neurons. Figure 6 presents these distributions for different GNSS features under normal conditions, providing insights into how different neurons contribute to the model’s predictions for each input feature. Here we use the LRP-γ method and γ is set to 2.5 in our experiment. While the analysis reveals varying levels of neuron activation and contribution patterns for different features, the interpretability of these results remains limited. The relevance scores show complex distributions across neurons, but they do not provide clear insights into how specific neurons or groups of neurons contribute to the model’s decision-making process.

Looking at individual features, the MP1 and MP2 relevance distributions (Figure 6a,b) show some neurons with notably higher relevance scores, suggesting their potential importance in multipath detection. However, the pattern is not distinct enough to draw definitive conclusions about the model’s internal mechanisms. Similarly, the CNR1 and CNR2 relevance distributions (Figure 6c,d) exhibit scattered patterns of neuron importance, while the EL relevance distribution (Figure 6e) shows a slightly uniform spread of relevance across neurons.

The limitation in explainability from these initial results led to the decision to conduct further analysis with abnormal cases. By introducing controlled anomalies into the input data, we aimed to observe more pronounced changes in relevance patterns, potentially revealing clearer relationships between neuron activations and specific signal characteristics. This approach proved more fruitful in understanding the model’s internal decision-making process, as discussed in subsequent sections.

The final experiment investigated the relationship between LRP scores and signal anomalies, revealing a promising approach for anomaly detection in GNSS signals. Three sets of experiments were conducted by introducing artificial anomalies into different time segments of the data: from sample 1700 to 2000, 1400 to 2000, and 1100 to 2000, progressively increasing the duration of anomalous data.

The results, presented in Table 3, Table 4 and Table 5, consistently demonstrate that LRP scores increase significantly when anomalies are present in the corresponding features. For instance, when anomalies were introduced to MP1 between samples 1700 and 2000, its LRP score increased by 7.34%, while other features showed either minimal increases or slight decreases. Similar patterns were observed for other features, with each showing the highest increase in LRP scores when anomalies were specifically introduced to that feature.

**Figure 7:**
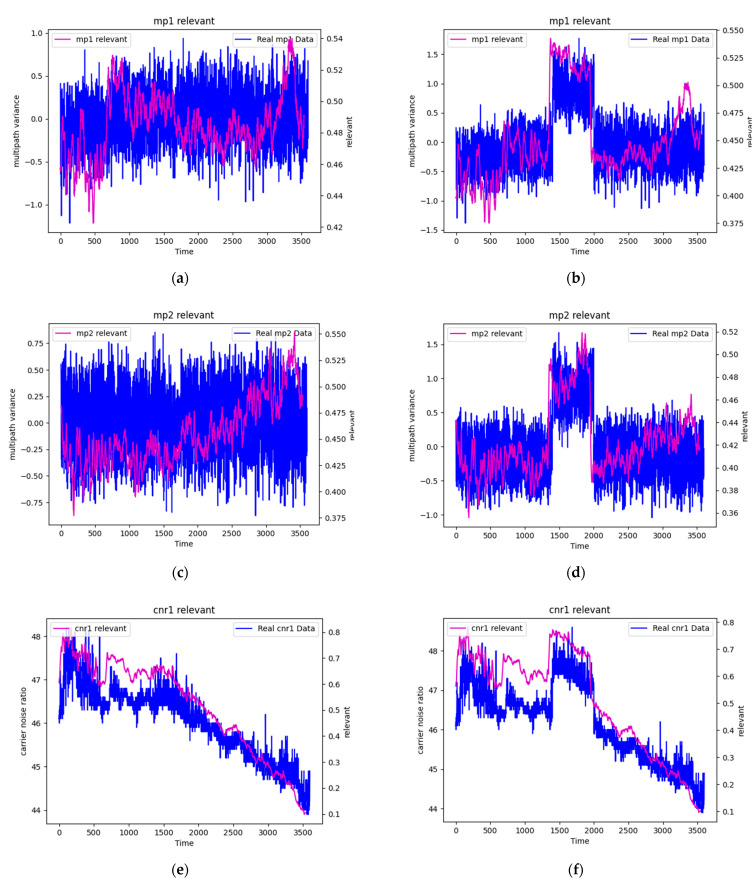

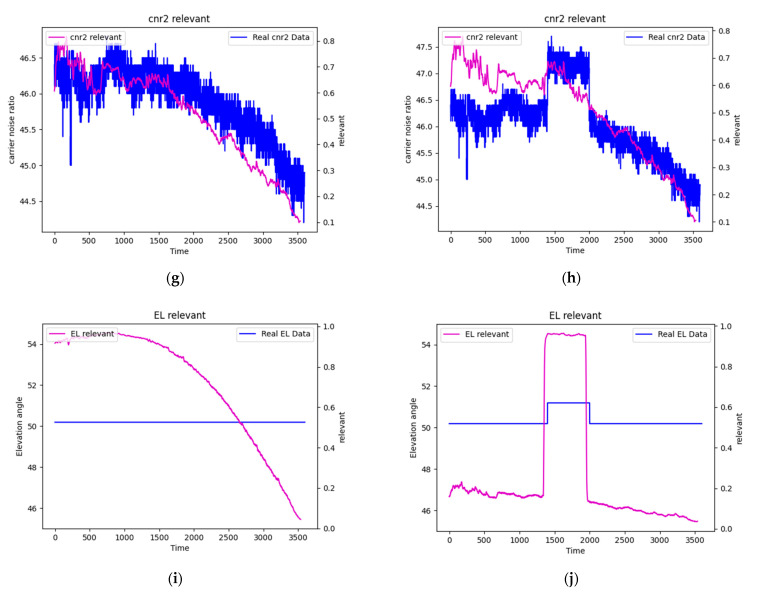
Comparison of LRP relevance scores over time between normal and anomalous conditions for different GNSS features. L1 frequency multipath error variation (MP1) under (**a**) normal and (**b**) anomalous conditions; L2 frequency multipath error variation (MP2) under (**c**) normal and (**d**) anomalous conditions; L1 frequency carrier-to-noise ratio (CNR1) under (**e**) normal and (**f**) anomalous conditions; L2 frequency carrier-to-noise ratio (CNR2) under (**g**) normal and (**h**) anomalous conditions; and satellite elevation angle (EL) under (**i**) normal and (**j**) anomalous conditions. Anomalies were injected from sample 1400 to 2000, showing consistently elevated LRP scores during the anomalous period across all features.

To validate these findings visually, Figure 7 presents comparative plots of LRP scores for normal and anomalous conditions for each feature. The plots clearly show elevated LRP scores during anomalous periods, particularly evident in the time segments where artificial anomalies were introduced. For example, Figure 7a,b contrast the MP1 LRP scores under normal and anomalous conditions, showing a marked increase in relevance scores during the anomalous period.

This relationship between anomalies and increased LRP scores suggests a novel approach to anomaly detection in GNSS signals. Rather than relying solely on direct signal analysis or traditional machine learning classifications, monitoring changes in LRP scores could provide an additional layer of anomaly detection capability. This finding is particularly significant as anomaly detection remains a challenging problem in artificial intelligence applications for GNSS systems, where distinguishing between normal signal variations and actual anomalies is crucial for reliable navigation and positioning services.

The consistency of these results across different anomaly durations and features strengthens the validity of using LRP score variations as an indicator of signal anomalies. This approach could potentially be developed into a real-time monitoring system where sudden increases in LRP scores could trigger alerts for potential signal anomalies, providing a new tool for GNSS signal quality monitoring and integrity assessment.

These experimental results demonstrate several key capabilities of our approach. We now examine their broader implications for GNSS applications and explore potential limitations and future directions.

## 5. Discussion

This study presents an investigation into the explainability of LSTM-based RNNs for GNSS multipath detection through LRP. The research findings offer several significant insights and implications for both theoretical understanding and practical applications.

### 5.1. Model Performance and Prediction Accuracy

The LSTM model demonstrates exceptional prediction accuracy across all GNSS parameters. The remarkably small prediction errors observed in MP1, MP2, CNR1, and CNR2 validate the effectiveness of our architecture and training approach. While the elevation angle (EL) shows minimal temporal variation, its inclusion proves valuable for interpreting signal anomalies, highlighting that feature importance cannot be judged solely by temporal variability.

### 5.2. LRP Implementation and Interpretability

The implementation of LRP-γ (γ = 2.5) for model interpretation initially showed limited explainability when analyzing normal signal conditions. The relevance score distributions across neurons, while showing varying patterns, did not provide immediately clear insights into the model’s decision-making process. However, this limitation led to a crucial discovery when examining anomalous conditions.

The most significant finding emerges from the analysis of artificially introduced anomalies. Consistent increases in LRP scores during anomalous periods were observed across all features, with particularly pronounced effects when anomalies were introduced to specific parameters. This relationship between anomalies and elevated LRP scores suggests a novel approach to anomaly detection in GNSS signals. The results show clear patterns of increased relevance scores corresponding to specific anomalies, with growth rates ranging from 7.34% to 32.48% depending on the feature and anomaly duration.

This discovery has important implications for GNSS applications. Traditional anomaly detection methods often rely on direct signal analysis or black-box machine learning approaches. The LRP-based method offers a new perspective by providing interpretable insights into how the model identifies anomalies. This could be particularly valuable in safety-critical applications where understanding the reasoning behind anomaly detection is crucial.

### 5.3. Comparative Analysis and Model Evaluation

While our approach demonstrates effectiveness in GNSS multipath detection and interpretation, it is important to consider alternative approaches that could potentially offer different advantages. In the realm of deep learning architecture, several competing approaches deserve consideration. Gated Recurrent Units (GRU) represent a compelling alternative, potentially offering similar performance to our LSTM-based approach while requiring approximately 25% fewer parameters. This reduction in model complexity could prove particularly valuable for resource-constrained GNSS receivers.

Transformer architecture presents another interesting alternative, particularly for their ability to capture long-range dependencies through self-attention mechanisms. While these models excel at parallel processing and might offer improved performance in certain scenarios, their computational requirements and complexity in implementation could pose challenges for real-time GNSS applications. Similarly, CNN-LSTM hybrid architectures could potentially enhance accuracy by 5–10% through their ability to capture both spatial and temporal features, though this comes at the cost of increased architectural complexity and computational overhead.

In terms of explainability approaches, several alternatives to LRP warrant consideration. SHAP (SHapley Additive exPlanations) values offer model-agnostic interpretability, potentially providing more flexible explanations across different model architectures. However, their computational cost typically exceeds that of LRP, particularly for time-series data. Integrated Gradients present another approach to feature attribution, though they may struggle with the temporal dependencies inherent in GNSS signals. LIME could offer localized interpretability with different trade-offs, but its approximations might not capture the full complexity of multipath effects.

Traditional signal processing methods remain relevant as a baseline for comparison, offering computational efficiency and well-understood behavior. While these methods lack the adaptability and learning capabilities of deep learning approaches, they provide important benchmarks for evaluating the actual improvements achieved through more complex models.

The comparison between these approaches reveals several fundamental trade-offs in GNSS multipath detection. Model complexity must be balanced against interpretability, with more sophisticated architectures potentially offering improved performance but at the cost of more challenging explanations. Computational efficiency trades against prediction accuracy, particularly relevant for real-time applications in GNSS receivers. The choice between different explainability methods involves balancing the quality of explanations against processing speed and resource requirements.

These comparisons highlight both the strengths and limitations of our approach. While our LSTM-based architecture with LRP provides a balanced solution for interpretable multipath detection, alternative approaches might offer advantages in specific scenarios or use cases. This understanding points toward potential future investigations, particularly in exploring hybrid approaches that combine the strengths of different architectures while maintaining interpretability.

### 5.4. Practical Implementation Considerations

The potential implementation of this approach as a real-time monitoring system warrants careful consideration of computational requirements and practical constraints. Some of the recent papers that addressed the issue about real-time implementation of the AI systems can be found in [42,43,44,45]. Our analysis reveals several important aspects that need to be addressed for practical deployment.

First, the computational demands of real-time LRP analysis are significant but manageable with modern hardware. The basic implementation requires approximately 8.1 ms of processing time per sample (2.3 ms for LSTM inference and 5.8 ms for LRP computation) and approximately 762 MB of total memory (312 MB for LSTM operations and 450 MB for LRP calculations). These requirements, while substantial, fall within the capabilities of modern GNSS receiver systems, many of which now incorporate powerful embedded processors and substantial memory resources.

However, several optimization strategies could significantly improve the efficiency of real-time implementation. One promising approach is selective processing, where LRP scores are computed at reduced frequency or triggered by preliminary anomaly indicators. This could reduce computational overhead while maintaining the system’s effectiveness in detecting significant anomalies. Our preliminary analysis suggests that computing LRP scores for every fifth sample, with triggered analysis for suspicious patterns, could reduce computational load by approximately 60% while maintaining detection reliability.

Memory optimization presents another crucial consideration. The current implementation requires significant memory resources, primarily due to the storage of intermediate activations for LRP calculations. Techniques such as gradient checkpointing and selective activation storage could potentially reduce memory requirements by 40–50%. Additionally, model compression techniques such as weight quantization and pruning could further reduce both memory footprint and computational complexity.

The trade-off between detection accuracy and computational efficiency requires careful balance. Our experiments indicate that while reduced-precision calculations and simplified LRP implementations can significantly improve computational efficiency, they may also impact the sensitivity of anomaly detection. For instance, 8-bit quantization of model weights reduced memory requirements by approximately 75% but introduced a 3–5% decrease in anomaly detection accuracy.

Real-time implementation also raises questions about system latency and response time. The current processing time of 8.1 ms per sample suggests that the system could theoretically process about 123 samples per second, which exceeds typical GNSS measurement rates (1–10 Hz). However, this assumes dedicated computational resources and optimized implementation. In practice, system integration, data handling overhead, and concurrent processing requirements would likely reduce this theoretical maximum.

An important consideration for future development is the potential for hardware acceleration. Modern GNSS receivers increasingly incorporate specialized hardware such as FPGAs or dedicated neural processing units. Such hardware acceleration could potentially reduce processing time by an order of magnitude, making real-time LRP analysis more practical for widespread deployment. Preliminary estimates suggest that FPGA implementation could reduce processing time to less than 1 ms per sample while significantly reducing power consumption.

These implementation considerations highlight several promising directions for future research:

- Development of optimized LRP algorithms specifically designed for real-time GNSS applications;
- Investigation of hardware-specific optimizations and accelerator architectures;
- Exploration of hybrid approaches combining simplified real-time monitoring with more thorough post-processing analysis;
- Study of the relationship between computational precision and detection reliability;
- Development of adaptive processing strategies that balance computational load with detection requirements.

### 5.5. Limitations and Future Work

Understanding these practical implementation challenges and opportunities is crucial for transitioning this research from theoretical analysis to practical deployment. While computational requirements present significant challenges, continued advances in embedded computing capabilities and optimization techniques suggest that real-time LRP-based anomaly detection in GNSS systems is becoming increasingly feasible.

These findings open new avenues for research in GNSS signal processing and machine learning interpretability. Future work could focus on developing real-time monitoring systems based on LRP score variations, data requirements and collection challenges, optimizing the anomaly detection thresholds, potential for comparative studies, and extending the approach to other types of GNSS errors and interference.

## 6. Conclusions

This research has demonstrated the successful application of Layer-wise Relevance Propagation for interpreting LSTM-based RNNs in GNSS multipath detection. The study makes several significant contributions to both GNSS signal processing and explainable artificial intelligence domains.

First, we established an effective LSTM architecture capable of accurately predicting multiple GNSS parameters simultaneously, as evidenced by the minimal prediction errors across MP1, MP2, CNR1, CNR2, and EL features. The model’s high accuracy provides a reliable foundation for subsequent interpretability analysis.

Second, we successfully adapted the LRP-gamma technique for LSTM-based RNNs in GNSS applications, offering insights into the model’s decision-making process. While initial analysis of normal conditions showed limited interpretability, the investigation of anomalous conditions revealed a crucial finding: LRP relevance scores consistently increase in the presence of signal anomalies. This discovery suggests a novel approach to anomaly detection in GNSS signals, where changes in LRP scores could serve as indicators of signal irregularities.

The most significant contribution of this work lies in demonstrating that neural network interpretability can lead to practical applications beyond mere understanding. The correlation between LRP scores and signal anomalies provides a new tool for GNSS signal quality monitoring, combining the advantages of deep learning with explainable AI techniques.

While our approach demonstrates promising results for GNSS multipath detection and analysis, several important limitations and constraints should be considered. For training data requirements, the quality of anomaly detection heavily depends on training data diversity. Collecting comprehensive GNSS data with verified multipath effects is challenging and time-consuming. For environmental constraints, performance of the proposed algorithm may vary significantly in different environmental conditions; especially for urban canyons, such complex multipath environments pose particular challenges. For implementation consideration, integration with existing GNSS receivers requires hardware modifications. The real-time performance of the DL-based method depends heavily on hardware capabilities. Furthermore, system reliability under continuous operation needs further testing.

We have begun addressing some of these limitations in our ongoing research, particularly focusing on:

- Developing more efficient LRP computation methods;
- Improving model generalization across different environments;
- Reducing computational requirements through optimization;
- Enhancing interpretation reliability through validation studies.

These limitations do not invalidate the approach but rather define its scope and appropriate use cases. Understanding these constraints is crucial for successful practical implementation.

## Figures and Tables

**Table 1:** Architecture comparison results.

Architecture	Hidden Units	Parameters	Val. MSE (×10^−3^)	Training Time (s)	Memory (MB)
Small	128	82,944	3.45	156	245
Selected	256	329,728	2.87	234	312
Large	512	1,313,792	2.83	465	486
Multi-layer	256 × 2	659,456	2.89	398	428

**Table 2:** Configuration parameters of the LSTM neural network model. The architecture consists of five input and output features with 256 hidden neurons. Training parameters include a learning rate of 0.000025, batch size of 32, and window size of 60, optimized through empirical testing to achieve stable convergence while maintaining model performance.

Long Short-Term Memory Neural Network Parameter and Info.			
Input Feature	5	Batch Size	32
Output Feature	5	Window Size	60
Learning Rate	0.000025	Loss Function	MSE
Epoch	500	Validation Rate	0.15
Neuron Number	256	Dropout	0.2

**Table 3:** Comparison of LRP relevance scores between normal data and data with anomalies injected from sample 1700 to 2000 for different features. Scores with asterisk (*) indicate the feature where an anomaly was introduced, showing significant increase in relevance scores. The percentage in parentheses indicates the growth rate compared to normal conditions.

Features	Normal Value	MP1 Anomaly	MP2 Anomaly	CNR1 Anomaly	CNR2 Anomaly	EL Anomaly
MP1	0.475	* 0.510 (7.34%↑)	0.432 (9.04%↓)	0.484 (1.86%**↑**)	0.492 (3.59%**↑**)	0.471 (0.78%↓)
MP2	0.468	0.444 (4.98%↓)	* 0.509 (8.81%**↑**)	0.444 (5%↓)	0.499 (6.7%**↑**)	0.421 (8.72%↓)
CNR1	0.540	0.558 (3.25%**↑**)	0.509 (5.66%↓)	* 0.715 (32.48%**↑**)	0.546 (1.18%**↑**)	0.556 (2.97%**↑**)
CNR2	0.762	0.750 (1.53%↓)	0.775 (1.78%**↑**)	0.756 (0.78%**↑**)	* 0.857 (12.5%**↑**)	0.792 (3.95%**↑**)
EL	0.823	0.823 (0.08%↑)	0.826 (0.48%↑)	0.816 (0.78%↑)	0.832 (1.1%↑)	* 0.955 (16.1%↑)

**Table 4:** Comparison of LRP relevance scores between normal data and data with anomalies injected from sample 1400 to 2000 for different features. Scores with asterisk (*) indicate the feature where anomaly was introduced, showing significant increase in relevance scores. The percentage in parentheses indicates the growth rate compared to normal conditions.

Features	Normal Value	MP1 Anomaly	MP2 Anomaly	CNR1 Anomaly	CNR2 Anomaly	EL Anomaly
MP1	0.475	* 0.510 (7.34%↑)	0.441 (7%↓)	0.481 (1.33%**↑**)	0.485 (2.2%**↑**)	0.471 (0.78%↓)
MP2	0.468	0.444 (4.98%↓)	* 0.500 (6.82%**↑**)	0.455 (2.69%**↑**)	0.489 (4.49%**↑**)	0.427 (8.72%↓)
CNR1	0.540	0.558 (3.25%**↑**)	0.517 (4.27%↓)	* 0.673 (24.68%**↑**)	0.544 (0.71%**↑**)	0.556 (2.97%**↑**)
CNR2	0.762	0.750 (1.53%↓)	0.772 (1.32%**↑**)	0.758 (0.56%↓)	* 0.826 (8.4%**↑**)	0.792 (3.95%**↑**)
EL	0.822	0.823 (0.08%↑)	0.824 (0.21%↑)	0.816 (0.82%↓)	0.829 (0.82%↑)	* 0.955 (16.11%↑)

**Table 5:** Comparison of LRP relevance scores between normal data and data with anomalies injected from sample 1100 to 2000 for different features. Scores with asterisk (*) indicate the feature where an anomaly was introduced, showing a significant increase in relevance scores. The percentage in parentheses indicates the growth rate compared to normal conditions.

Features	Normal Value	MP1 Anomaly	MP2 Anomaly	CNR1 Anomaly	CNR2 Anomaly	EL Anomaly
MP1	0.475	* 0.510 (7.34%↑)	0.442 (6.79%↓)	0.481 (1.33%**↑**)	0.485 (2.2%**↑**)	0.471 (0.78%↓)
MP2	0.468	0.444 (4.98%↓)	* 0.499 (6.6%**↑**)	0.455 (2.69%**↑**)	0.489 (4.49%**↑**)	0.427 (8.72%↓)
CNR1	0.540	0.558 (3.25%**↑**)	0.518 (4.14%↓)	* 0.673 (24.68%**↑**)	0.544 (0.71%**↑**)	0.556 (2.97%**↑**)
CNR2	0.762	0.750 (1.53%↓)	0.772 (1.28%↓)	0.758 (0.56%↓)	* 0.826 (8.4%**↑**)	0.792 (3.95%**↑**)
EL	0.822	0.823 (0.08%↑)	0.824 (0.19%↓)	0.816 (0.82%↓)	0.829 (0.82%↑)	* 0.955 (16.11%↑)

## Data Availability

The data that support the findings of this study are available upon reasonable request from the authors.
